# 4.1Ba is necessary for glutamatergic synapse formation in the sensorimotor circuit of developing zebrafish

**DOI:** 10.1371/journal.pone.0205255

**Published:** 2018-10-04

**Authors:** Javier Fierro, Dylan R. Haynes, Philip Washbourne

**Affiliations:** Institute of Neuroscience, University of Oregon, Eugene, Oregon, United States of America; University of Queensland, AUSTRALIA

## Abstract

During the process of synapse formation, thousands of proteins assemble at prospective sites of cell-cell communication. Although many of these proteins have been identified, the roles they play in generating functional connections during development remain unknown. 4.1 scaffolding proteins have been implicated in synapse formation and maturation *in vitro*, but *in vivo* studies for some family members have suggested these proteins are not important for this role. We examined the role of family member 4.1B because it has been implicated in glutamatergic synaptogenesis, but has not been described *in vivo*. We identified two *4*.*1B* genes in zebrafish, *4*.*1Ba* and *4*.*1Bb*, by sequence comparisons and synteny analysis. *In situ* hybridization shows these genes are differentially expressed, with *4*.*1Ba* expressed primarily in the nervous system and *4*.*1Bb* expressed in the nervous system and muscle, but not the spinal cord. We focused our studies on 4.1Ba in the spinal cord. *4*.*1Ba* knockdown reduced the number of glutamatergic synapses at caudal primary motor neurons and caused an increase in the duration of touch-evoked coiling. These results suggest 4.1Ba is important for the formation of functional glutamatergic synapses in the developing zebrafish spinal cord.

## Introduction

The central nervous system (CNS) is composed of trillions of synapses, important cellular structures that together coordinate the multitude of functions of the brain. Each of these cell-cell junctions is assembled from thousands of different synaptic proteins important for establishing, operating, and maintaining these synapses over time [1]. Genetic mutations that disrupt these proteins can change how the macromolecular complexes form and function at the synapse, ultimately leading to neurological disorders such as autism and schizophrenia [2, 3]. To further our understanding of the mechanisms that underlie these disorders, a complete characterization of the synaptic proteins involved in normal brain activity is necessary. In the present study, we investigated the role of protein 4.1B during neuronal development in zebrafish (*Danio rerio*) embryos.

4.1B is a cytoplasmic scaffolding molecule involved in stabilizing macromolecular complexes at cell membranes. 4.1B belongs to the family of 4.1 proteins that includes 4.1R, 4.1N, and 4.1G (reviewed in [4]). Although all 4.1 proteins are broadly expressed, 4.1B shows strong expression within the mammalian CNS [5], and is enriched in postsynaptic densities [6]. 4.1 proteins are characterized by three domains: the Four-point-one, Ezrin, Radixin, and Moesin (FERM) domain, the Spectrin-Actin Binding (SAB) domain, and the C-Terminal Domain (CTD) [7–9]. The FERM domain, whose activities are regulated by the FERM-Adjacent (FA) region [10], binds to cell adhesion molecules (CAMs) [11], Membrane Associated Guanylate Kinase (MAGUK) proteins [12], and the phospholipid bilayer [13]. The SAB domain tethers 4.1B to the actin cytoskeleton [7] and is a specialization of vertebrates that enhances the stability of protein complexes at the submembranous cytoskeleton [14]. Lastly, the CTD interacts with neurotransmitter receptors. The CTD of 4.1N and 4.1G interacts with α-amino-3-hydroxy-5-methyl-4-isoxazolepropionic acid (AMPA) receptors [15], stabilizing them at the submembranous cytoskeleton *in vitro*. Although it is unclear whether there is a direct interaction, 4.1B can enhance the recruitment and stabilization of N-methyl D-aspartate (NMDA) receptors in neuronal cultures [16]. The localization of 4.1B at the postsynaptic density, and the numerous interactions between 4.1B and other synaptic and submembranous elements, suggests 4.1B may play an important role in the postsynaptic specialization for establishing and maintaining functional glutamatergic synapses.

Interestingly, knockdown studies of 4.1N and 4.1G in mice have shown no effect on synaptic structure or number, and have shown no effect on AMPA or NMDA receptor mediated transmission in hippocampal neurons [17]. These data suggest those 4.1 proteins are not important for synaptic function despite their interactions with synaptic proteins and their localization at the postsynaptic density.

To identify a role for 4.1B at synapses *in vivo*, we characterized 4.1B in the spinal cord of developing zebrafish embryos. At early developmental stages, zebrafish exhibit few synapses in the spinal cord, allowing for easy quantification of synapse number on single neurons in the developing animal [18]. At 26 hours post fertilization (hpf), zebrafish exhibit two simple behaviors controlled by circuits within the spinal cord: spontaneous contractions dependent on electrical synapses and touch-evoked coiling dependent on electrical and chemical synapses [19]. The circuit underlying touch-evoked coiling is well characterized and is reliant on glutamatergic synapses [20] that can be individually identified [18]. These characteristics make the developing spinal cord of zebrafish an ideal model for studying the influence of 4.1B on glutamatergic synapses and their function.

We identified two differentially expressed *4*.*1B* orthologs encoded by the genes *epb41l3a* and *epb41l3b* in zebrafish. For clarity, we will refer to the genes as *4*.*1Ba* and *4*.*1Bb*, respectively, for the remainder of this paper. *4*.*1Ba* is expressed exclusively in the brain and spinal cord, while *4*.*1Bb* is expressed in the brain and myotome. We studied the role of 4.1Ba at synapses in the spinal cord responsible for mediating touch-evoked behaviors. Knockdown studies of *4*.*1Ba* showed a reduction in the number of glutamatergic synapses at motor neurons, and an overall increase in the duration of touch-evoked coiling at 26 hpf. These results suggest 4.1Ba is important for the formation and maturation of glutamatergic synapses in the developing zebrafish spinal cord.

## Results

### Characterization of zebrafish *4*.*1B* homologues

**Fig 1:**
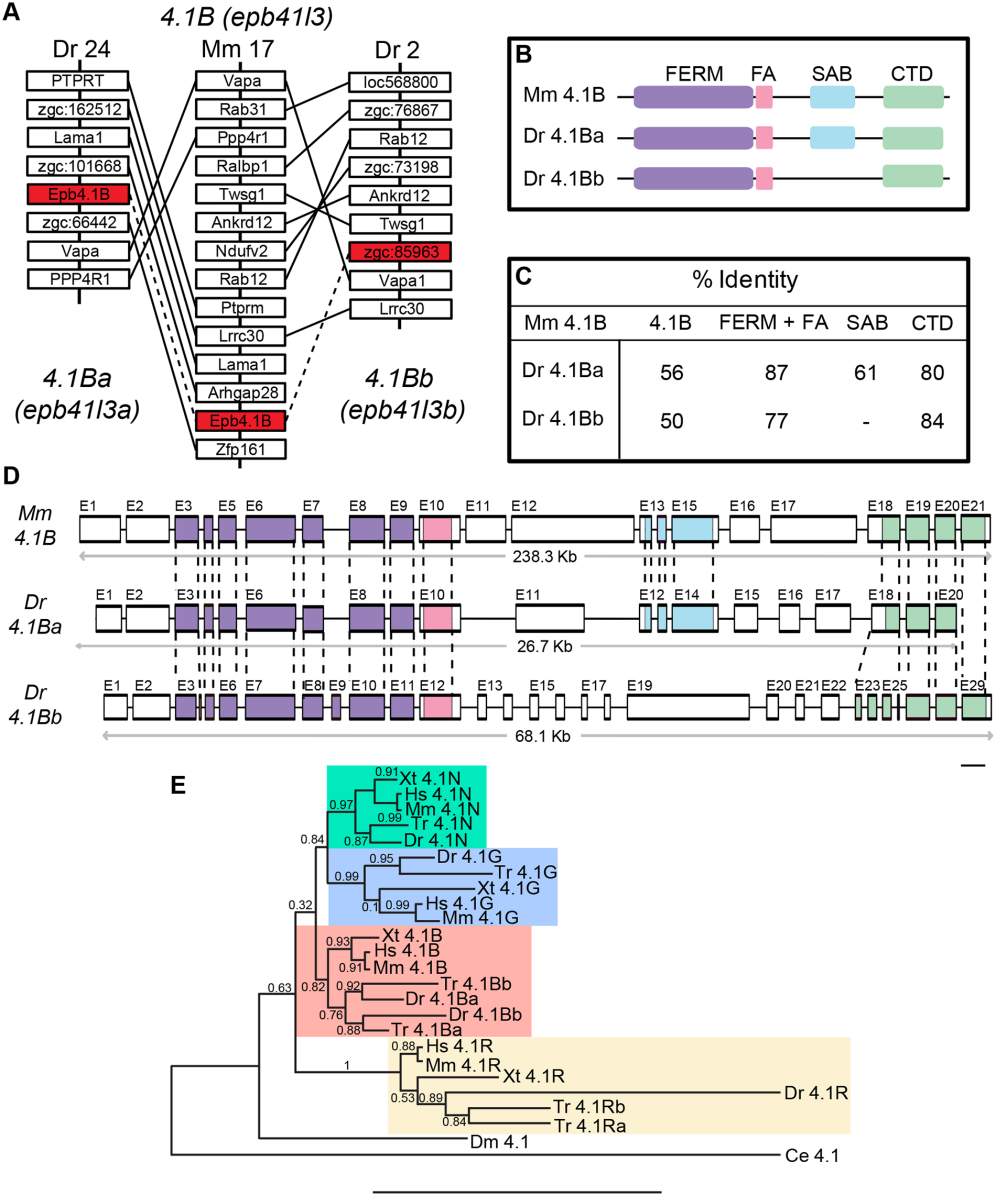
4.1B characterization in zebrafish. (**A**) Synteny analysis of duplicated *4*.*1B* genes in the zebrafish *Danio rerio* (Dr). Red boxes connected by dashed lines indicate orthologous Dr *4*.*1B* genes with mouse (Mm) *4*.*1B*. *4*.*1B* on Dr linkage group 24 has been named *4*.*1Ba*. Distances between genes are not drawn to scale. (**B**) Schematic representation of 4.1B structure. Purple box—FERM Domain; Pink box—FA region; Blue box—SAB domain; Green Box—CTD. Only 4.1Ba contains an SAB domain, analogous to mammalian 4.1B. (**C**) Pairwise alignments of Dr 4.1B and its individual domains compared to Mm 4.1B. Homology between species is represented as a percentage of amino acid identity. Although there appears to be low homology between species, individual domains of Dr 4.1B are highly homologous to Mm 4.1B domains. (**D**) Genomic analysis of *4*.*1B* genes. Individual exon analysis revealed strong conservation of genomic structure between species. Colored exons represent protein coding regions for the domains with the same color code as in B. Exons are drawn to scale; scale bar: 100 bp. (**E**) Phylogenetic analysis of 4.1 proteins in various invertebrate and vertebrate animals. Bootstrap analysis suggests Dr 4.1B proteins are more closely related to vertebrate 4.1B proteins than to other 4.1 family members. The branch points are the percentage of bootstrap values for 1,000 replicas. Scale bar: 0.5 substitutions per site. Ce = *Caenorhabditis elegans*, Dm = *Drosophila melanogaster*, Hs = *Homo sapiens*, Tr = *Takifugu rubripes*, Xt = *Xenopus tropicalis*.

4.1 proteins are highly expressed in the brain [5], yet their roles in CNS development and function have not been well examined. We chose to study 4.1B in the zebrafish embryo because it is an ideal model for the analysis of gene function during synapse development [18]. We identified two *4*.*1B* genes in the zebrafish genome, *4*.*1Ba* (*epb41l3a*) and *4*.*1Bb* (*epb41l3b)*, using mouse *4*.*1B* (*epb41l3*) as our template (Fig 1A). Given the sequence similarity between 4.1 family members, we performed an analysis of conserved synteny to ensure the identified genes are co-orthologs of mammalian *4*.*1B*, i.e. duplicates of the single mammalian ortholog that arose from a genome duplication event [21]. This analysis revealed the genes surrounding the putative *4*.*1B* genes on zebrafish linkage groups 24 and 2 are homologous to the genes surrounding mouse *4*.*1B* on chromosome 17 (Fig 1A), demonstrating the identified *4*.*1B* genes are both orthologous with mammalian *4*.*1B*.

Pairwise alignments of the zebrafish 4.1B protein sequences with mouse 4.1B revealed both predicted protein sequences contain a FERM domain, an FA region, and the CTD (Fig 1B). Only 4.1Ba possesses an SAB domain, suggesting 4.1Ba is more conserved with mouse 4.1B. Overall amino acid composition of mouse 4.1B is 56% conserved with 4.1Ba, and 50% conserved with 4.1Bb (Fig 1C). Despite relatively low conservation, these proteins are highly conserved within their structural domains. For example, the FERM domain and the FA region together are 87% conserved in 4.1Ba, and 77% conserved in 4.1Bb (Fig 1C). This level of conservation suggests the function of the individual domains are well preserved. The lack of the SAB domain in 4.1Bb suggests the two 4.1B proteins in zebrafish may have evolved divergent functions.

We next compared the genomic organization of mouse and zebrafish *4*.*1B* genes to further examine evolutionary events that may have modified these genes over time (Fig 1D). We noticed considerable conservation of exon structure between the mouse and zebrafish genes. For example, the FA region is translated from a single exon in all genes (Mouse *4*.*1B* and Zebrafish *4*.*1Ba*: exon 10; Zebrafish *4*.*1Bb*: exon 12). Sequence similar to the SAB domain in mouse *4*.*1B* (exons 13–15) was found in *4*.*1Ba* (exons 12–14), but not in *4*.*1Bb*, further supporting the conclusion 4.1Bb does not contain an SAB domain. These analyses suggest that, despite minor structural differences, *4*.*1Ba* and *4*.*1Bb* are well conserved with their mammalian ortholog.

We next conducted a phylogenetic analysis to confirm the relationship between various invertebrate and vertebrate 4.1 family members [22] (Fig 1E). Our analysis revealed the identified zebrafish 4.1B proteins are most closely related to all other vertebrate 4.1B proteins, further providing evidence for evolutionary conservation of these proteins. We note that zebrafish 4.1Ba is more closely related to pufferfish (*Tr*, *Takifugu rubripes*) 4.1Bb in our phylogenetic analysis. This is a discrepancy in name designation and does not reflect a structural difference.

### *4*.*1Ba* and *4*.*1Bb* are differentially expressed within the developing embryo

**Fig 2:**
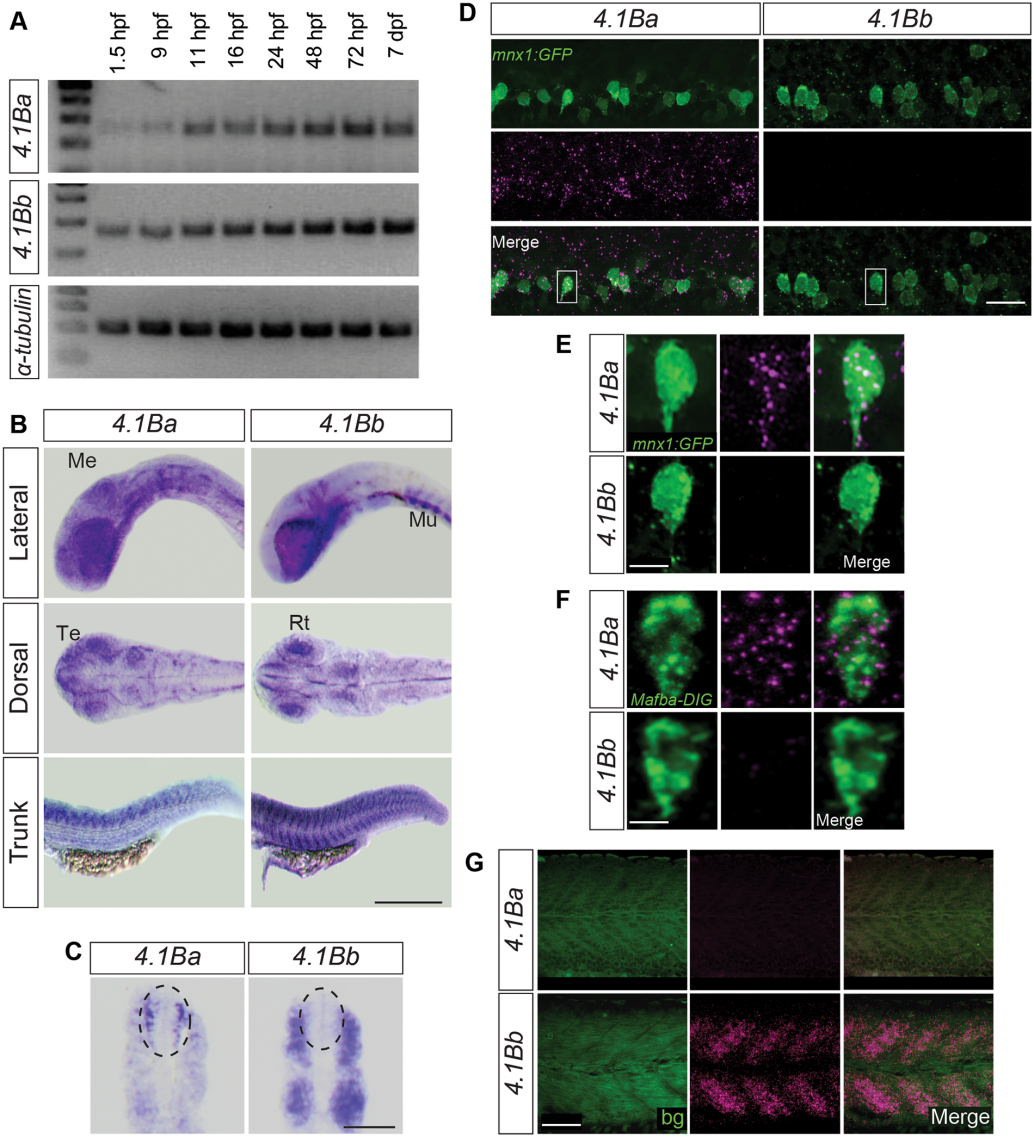
Duplicated *4*.*1B* genes are differentially expressed in the zebrafish trunk. (**A**) Analysis of *4*.*1B* expression levels during various stages of development. *4*.*1Ba* and *4*.*1Bb* are expressed throughout development. From left to right, cDNA samples were derived from wild-type (AB/Tübingen) zebrafish at the 16-cell (1.5 hpf), 90% epiboly (9 hpf), 3-somite (11 hpf), 16-, 24-, 48-, 72 hpf, and 7 days post fertilization (dpf) stages. (**B**) *in situ* hybridization showing *4*.*1Ba* and *4*.*1Bb* expression in the brain and trunk of the developing zebrafish embryo at 26 hpf. Both *4*.*1Ba* and *4*.*1Bb* are expressed in the telencephalon (Te), the mesencephalon (Me), and retina (Rt). Only *4*.*1Bb* was seen in muscle (Mu). (**C**) Cross section of the trunk at 26 hpf further demonstrates only *4*.*1Ba* is expressed in the spinal cord (dotted ellipse), whereas *4*.*1Bb* is expressed in muscle. (**D—G**) Fluorescent *in situ* hybridization of the trunk confirming expression of *4*.*1Ba*, but not *4*.*1Bb* in CaPs (**E**) and CoPA cells (**F)**, and *4*.*1Bb*, but not *4*.*1Ba*, in muscle (**G**) at 26 hpf. White boxes in D indicate cells shown in E. Scale Bar in B: 500 μm; C: 100 μm; D: 20 μm; E, F: 5 μm; G: 50 μm; Abbreviations: bg = background from chicken anti-GFP antibody.

We next examined the temporal expression of the duplicated *4*.*1B* genes during embryonic development. RT-PCR analysis at various developmental stages suggests both genes are expressed at all stages of development (Fig 2A).

To determine the spatial expression of *4*.*1B* genes in the developing zebrafish, we performed whole-mount *in situ* hybridization at 26 hpf. *In situ* hybridization analysis revealed *4*.*1Ba* and *4*.*1Bb* are expressed within the brain, specifically within the telencephalon, mesencephalon, and retina. *4*.*1Ba* is expressed within the spinal cord, with strongest expression in the anterior spinal cord that gradually decreased caudally, consistent with the rostro-caudal gradient of spinal cord development (Fig 2B). In contrast, *4*.*1Bb* expression in the trunk was localized to the myotome at all ages examined (see cross sections, Fig 2C).

To determine the expression pattern of *4*.*1Ba* and *4*.*1Bb* in the spinal cord at cellular resolution, we performed fluorescent *in situ* hybridization (FISH). We analyzed the expression patterns at 26 hpf in tg(*mnx1*:*Gal4;UAS*:*GFP*) embryos to identify motor neurons. *mnx1* encodes a transcription factor expressed within motor neurons [23]. We chose to determine the expression patterns at primary motor neurons (PMNs), specifically caudal ascending PMNs (CaPs), because they mediate the first behaviors of the zebrafish, namely spontaneous contractions and touch-evoked coiling [24], and are easily identifiable due to their long descending axon through the ventral myotome [25]. We confirmed expression of *4*.*1Ba* throughout the spinal cord (Fig 2D), with specific expression in 100% of CaPs, with 20% of total *4*.*1Ba* mRNA signal located in GFP positive motor neurons (within segments 8–12; Fig 2D and 2E). *4*.*1Ba* was also expressed in spinal cord interneurons, suggesting *4*.*1Ba* may be present in other cells important for touch-evoked behaviors. We examined the expression pattern of *4*.*1Ba* within Commissural Primary Ascending (CoPA) interneurons, cells involved in the touch response neuronal circuit, by co-labeling with a probe to the *mafba* gene. This gene has been shown to be expressed in CoPA cells [26]. We found *4*.*1Ba* mRNA colocalized with 87% of *mafba* positive cells (comprising 11% of total *4*.*1Ba* mRNA expression in all labeled cells within segments 8–12), signifying *4*.*1Ba* is expressed in CoPA cells (Fig 2F). There was no detectable expression of *4*.*1Bb* in CaPs or in any other neuronal population within the spinal cord (Fig 2D–2F). We confirmed the presence of *4*.*1Bb* in the muscle, with no detectable expression of *4*.*1Ba* (Fig 2G). We conclude *4*.*1Ba* and *4*.*1Bb* are differentially expressed within the trunk of the animal, further strengthening the idea that these genes have evolved divergent functions.

### 4.1Ba is necessary for correct synapse number at motor neurons

**Fig 3:**
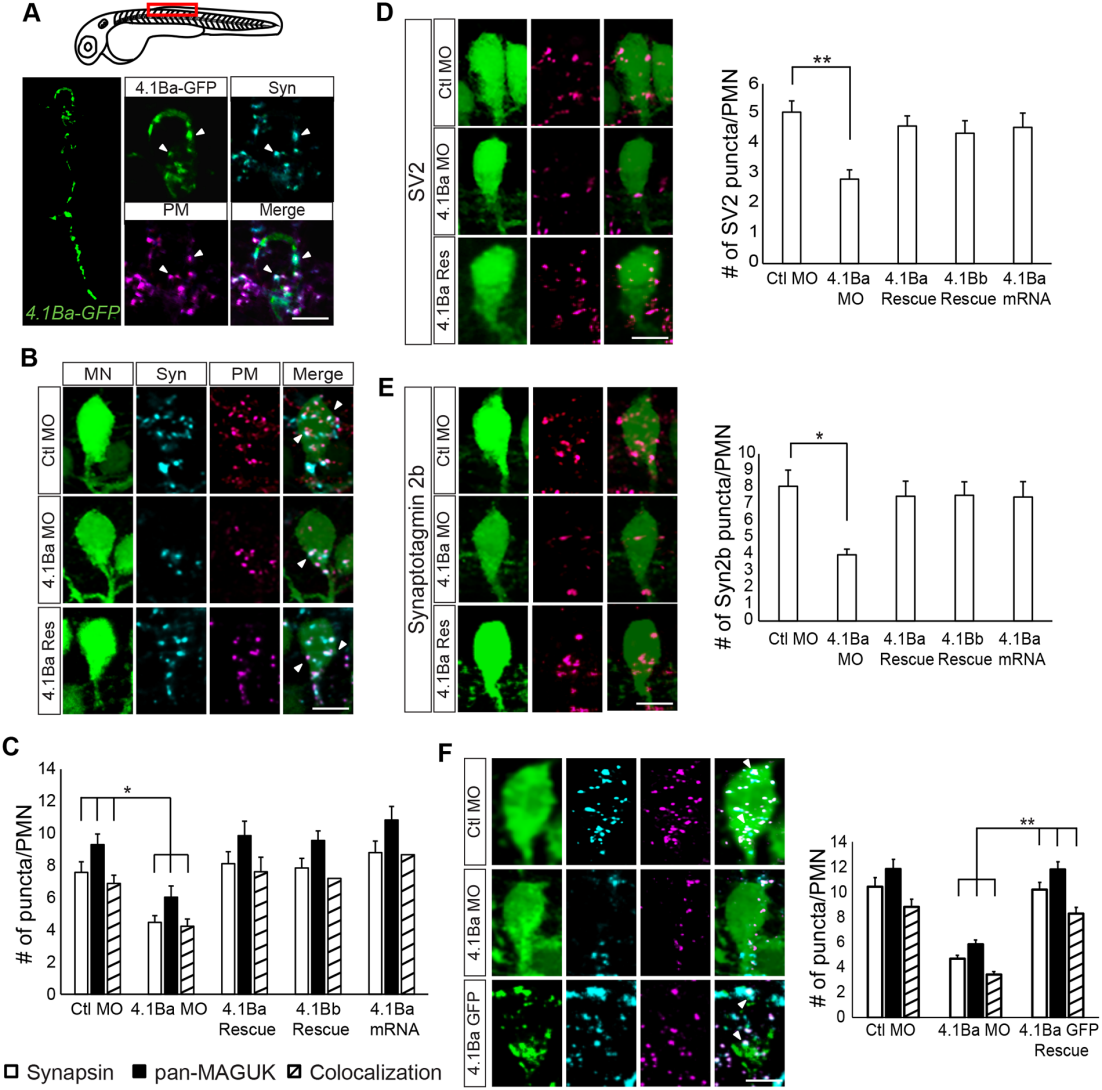
Knockdown of *4*.*1Ba* causes a reduction in the number of synapses at CaPs. (**A**) Labeling of 4.1Ba-GFP puncta colocalized with the presynaptic marker Synapsin 1/2 (Syn) and the postsynaptic marker panMAGUK (PM) at CaPs. Arrows indicate sites of colocalization. (**B**) Representative images of Syn and PM in Ctl MO, *4*.*1Ba* MO, and *4*.*1Ba* MO + *4*.*1Ba* RNA (4.1Ba Res) injected embryos. (**C**) Knockdown of *4*.*1Ba* reduced the number of synapses at CaPs. The phenotype was rescued with *4*.*1Ba* and *4*.*1Bb* mRNA (Rescue). Misexpression of *4*.*1Ba* did not affect synapse number. (**D—E**) Quantification of SV2 puncta (**D**) and Synaptotagmin 2b puncta (**E**) at CaPs. Both presynaptic proteins are reduced in the absence of *4*.*1Ba*. (**F**) Cell specific rescue of *4*.*1Ba* in CaPs. Embryos were collected from a tg(*mnx1*:*Gal4*:*UAS*:*GFP*) and tg(*mnx1*:*Gal4*) cross and injected with the Ctl MO, *4*.*1Ba* MO, or the *4*.*1Ba* MO + *UAS*:*4*.*1Ba-GFP* plasmid. Embryos were analyzed based on GFP expression. Expression of 4.1Ba-GFP restored the number of synapses specifically in CaPs. N = 10 for all conditions and experiments. Significance was evaluated with a One-way ANOVA followed by Tukey’s post-hoc analysis. All experiments were repeated in triplicate. Error bars represent s.e.m. * p < 0.05; ** p< 0.01. Scale bars in A—B, D—F: 5 μm. Abbreviations: Ctl MO = control morpholino; 4.1Ba MO = *4*.*1Ba* morpholino; 4.1Ba Rescue = *4*.*1Ba* MO *+ 4*.*1Ba* mRNA; 4.1Bb Rescue = *4*.*1Ba* MO + *4*.*1Bb* mRNA; 4.1Ba GFP Rescue = *4*.*1Ba* MO + *UAS*:*4*.*1Ba GFP* DNA.

Mammalian 4.1B has been isolated in biochemical postsynaptic density (PSD) preparations from pig and rat forebrain [6]. To determine whether 4.1Ba was localized to synapses in CaPs, we injected tg(*mnx1*:*Gal4*) embryos with a plasmid encoding *UAS*:*4*.*1Ba-GFP* and examined the spinal cord at the level of myotomes 8–12. The *4*.*1Ba* transgene was expressed with a punctate distribution in the cell body and axon of CaPs, suggesting protein 4.1Ba localizes to both pre- and postsynaptic specializations in motor neurons. To confirm a postsynaptic localization for 4.1Ba, we labeled embryos with antibodies to the presynaptic protein Synapsin 1/2, and to the postsynaptic proteins PSD-93, PSD-95, and Sap-97A (panMAGUK) [27] at 26 hpf (Fig 3A). We previously demonstrated that colocalization of these markers reveals the location of glutamatergic synapses in the developing spinal cord [18]. At this time of development most neurons have not yet developed extensive dendrites, and most afferent synapses are localized at cell bodies [18]. On CaP cell bodies, 91% of Synapsin 1/2 (Syn) puncta colocalized with panMAGUK (PM), while 78% of panMAGUK puncta colocalized with Synapsin 1/2, suggesting that the majority of puncta visualized with both antibodies are glutamatergic synapses. We found 4.1Ba-GFP colocalized with 82% of panMAGUK and Synapsin 1/2 positive puncta and is thus primarily localized at glutamatergic synapses.

To determine whether 4.1Ba is involved in the formation of these synapses, we knocked-down *4*.*1Ba* in tg(*mnx1*:*Gal4;UAS*:*GFP*) zebrafish embryos using a splice-site directed MO. This MO blocks the removal of intron 1, causing an early stop codon within the intronic sequence of the mRNA transcript (Part A in S1 Fig). Translation of this transcript leads to a truncated, nonfunctional protein. The specificity of knockdown was determined by extensive controls detailed in ‘*4*.*1Ba* and *4*.*1Bb* Morpholino Knockdown’ in the Methods and Materials section (see also parts A—C in S1 Fig), and were rigorously repeated every time the experiments were performed [28]. The *4*.*1Ba* MO caused an 86% reduction in correctly spliced *4*.*1Ba* mRNA transcripts.

*4*.*1Ba* knockdown caused a significant decrease in the number of Synapsin 1/2 and panMAGUK puncta at CaPs compared to control morpholino (Ctl MO) injected embryos (Syn—white bars: 41% decrease, p = 0.04; PM—black bars: 35% decrease, p = 0.017; Fig 3B and 3C). This decrease in pre- and postsynaptic markers led to an overall decrease in the number of colocalized puncta, or synapses, at CaPs (striped bars: 38% decrease, p = 0.041). This effect was rescued by coinjecting mRNA encoding zebrafish *4*.*1Ba* (4.1Ba Rescue, Fig 3B and 3C) as well as by *4*.*1Bb* mRNA (4.1Bb Rescue) which lacks the SAB domain (Fig 3C). This suggests the SAB domain may not be necessary for the formation of synapses at CaPs. No effect was seen when *4*.*1Ba* was overexpressed (4.1Ba mRNA, Fig 3C). Finally, we examined whether this effect was evident across all MNs. Knockdown of *4*.*1Ba* significantly reduced synapse number on all MNs (Ctl: 5.2 ± 0.24 synapses per cell versus *4*.*1Ba* MO: 3.6 ± 0.17, p = 1.2e-7). These experiments suggest 4.1Ba is necessary, but not sufficient, for the development of synapses in PMNs.

To date, studies of 4.1 protein function have focused solely on postsynaptic protein recruitment and stabilization. To characterize the presynaptic phenotype further, we examined the localization of two other presynaptic proteins, Synaptic Vesicle Protein 2 (SV2; Fig 3D) and Synaptotagmin 2b (Fig 3E), both transmembrane synaptic vesicle proteins. We found a 44% decrease in the number of SV2 puncta (p = 0.001) and a 51% decrease in Synaptotagmin 2b puncta (p = 0.037) at CaPs in *4*.*1Ba* knockdown animals. Both phenotypes were rescued by coinjecting *4*.*1Ba* or *4*.*1Bb* mRNAs (Fig 3D and 3E). We found no difference with misexpression of *4*.*1Ba*. These results suggest 4.1Ba is necessary, but not sufficient, for the formation or maintenance of presynaptic and postsynaptic terminals.

### Postsynaptic 4.1Ba instructs presynaptic assembly

As *4*.*1Ba* is expressed throughout the spinal cord, i.e. in other neurons synapsing onto motor neurons, we next examined whether the phenotype associated with *4*.*1Ba* knockdown is due to a cell autonomous or non-cell autonomous effect. Cell-specific rescue was achieved by injecting embryos from a cross between tg(*mnx1*:*Gal4*:*UAS*:*GFP*) and tg(*mnx1*:*Gal4*) lines with Ctl MO, *4*.*1Ba* MO, or *4*.*1Ba* MO and the *UAS*:*4*.*1Ba-GFP* plasmid. This resulted in animals with either mosaic expression of the transgenic 4.1Ba-GFP or expression of GFP in all motor neurons. We compared the synapse distribution between CaPs mosaically-expressing transgenic 4.1Ba-GFP in *4*.*1Ba* rescue animals and CaPs in *4*.*1Ba* MO and Ctl MO injected animals expressing GFP in all motor neurons. Expression of 4.1Ba-GFP resulted in a rescue of panMAGUK puncta at CaPs (black bars, Fig 3F), suggesting postsynaptic puncta number is regulated cell-autonomously by 4.1Ba. The number of Synapsin 1/2 puncta, which reside in presynaptic neurons not expressing the *UAS*:*4*.*1Ba-GFP* transgene, was also rescued, suggesting postsynaptic 4.1Ba is capable of transmitting a retrograde signal to recruit and/or maintain presynaptic components. This non-cell autonomous effect demonstrates 4.1Ba activity is necessary in the postsynaptic cell to form complete glutamatergic synapses at CaPs.

### 4.1Ba acts specifically in motor neurons on glutamatergic synapses

To determine whether the decrease in synapse number is specific to PMNs, we examined CoPA interneurons. These neurons are important for transmitting the sensory signals during the early sensorimotor response [20]. Analysis of Synapsin 1/2 and panMAGUK puncta revealed no differences in the number of puncta or synapses at these cells (Parts D and E in S1 Fig), suggesting 4.1Ba has different functions dependent on the cell type it is expressed in.

Whereas *4*.*1Ba* is expressed throughout the spinal cord, *4*.*1Bb* was not detected (Fig 2D–2F). As a further control for determining the specificity of the *4*.*1Ba* MO, we examined the effect of *4*.*1Bb* knockdown on synapse number in CaPs. Knockdown of *4*.*1Bb* with a splice blocking MO resulted in an 84% reduction in correctly spliced *4*.*1Bb* transcripts, but did not cause any changes in the number of Synapsin 1/2 or panMAGUK puncta at CaPs. The phenotype was not enhanced when the *4*.*1Bb* MO was coinjected with the *4*.*1Ba* MO (Parts F and G in S1 Fig). We saw no effect on synapse number when *4*.*1Bb* mRNA was misexpressed (Part G in S1 Fig). Altered morphology of muscle cells was observed when labeled with phalloidin after *4*.*1Bb* knock-down, consistent with its expression pattern (Fig 2G), but this phenotype was not further analyzed.

To examine whether the effect of *4*.*1Ba* knockdown was specific to glutamatergic synapses, we labeled inhibitory synapses in tg(*mnx1*:*Gal4;UAS*:*GFP*) embryos with antibodies to the protein Gephyrin (Parts H and I in S1 Fig). Gephyrin mediates the recruitment and stabilization of glycine receptors and GABA receptors at inhibitory synapses [29]. We found no decrease in the number of Gephyrin puncta at CaPs, confirming 4.1Ba is specifically involved in the formation of glutamatergic synapses.

### 4.1Ba is necessary for efficient touch-evoked coiling

Our results suggest 4.1Ba regulates the number of synapses onto CaPs at 26 hpf. At this developmental stage, zebrafish embryos have already developed the sensorimotor circuit which is dependent on the innervation of motor neurons, including CaPs, onto the ventral myotome [25]. It is therefore reasonable to think that loss of synapses onto PMNs, and in particular CaPs, due to *4*.*1Ba* knockdown may affect touch-evoked coiling. We first ensured *4*.*1Ba* knockdown did not affect CaP axon or muscle development. We saw no structural differences in CaPs between Ctl MO and *4*.*1Ba* MO injected embryos (Parts A—D in S2 Fig). We also found no differences in the structure or organization of the muscle, analyzed by immunolabeling with Phalloidin (Part A in S2 Fig). Finally, we examined acetylcholine receptor clusters at the neuromuscular junction (NMJ) by labeling with α-bungarotoxin. We found no differences in the size and number of NMJs between Ctl MO and *4*.*1Ba* MO-injected embryos (Parts A and E in S2 Fig). Together, these results show muscle and NMJ integrity is not affected by the absence of 4.1Ba, despite presynaptic localization in motor axons (Fig 3A).

**Fig 4:**
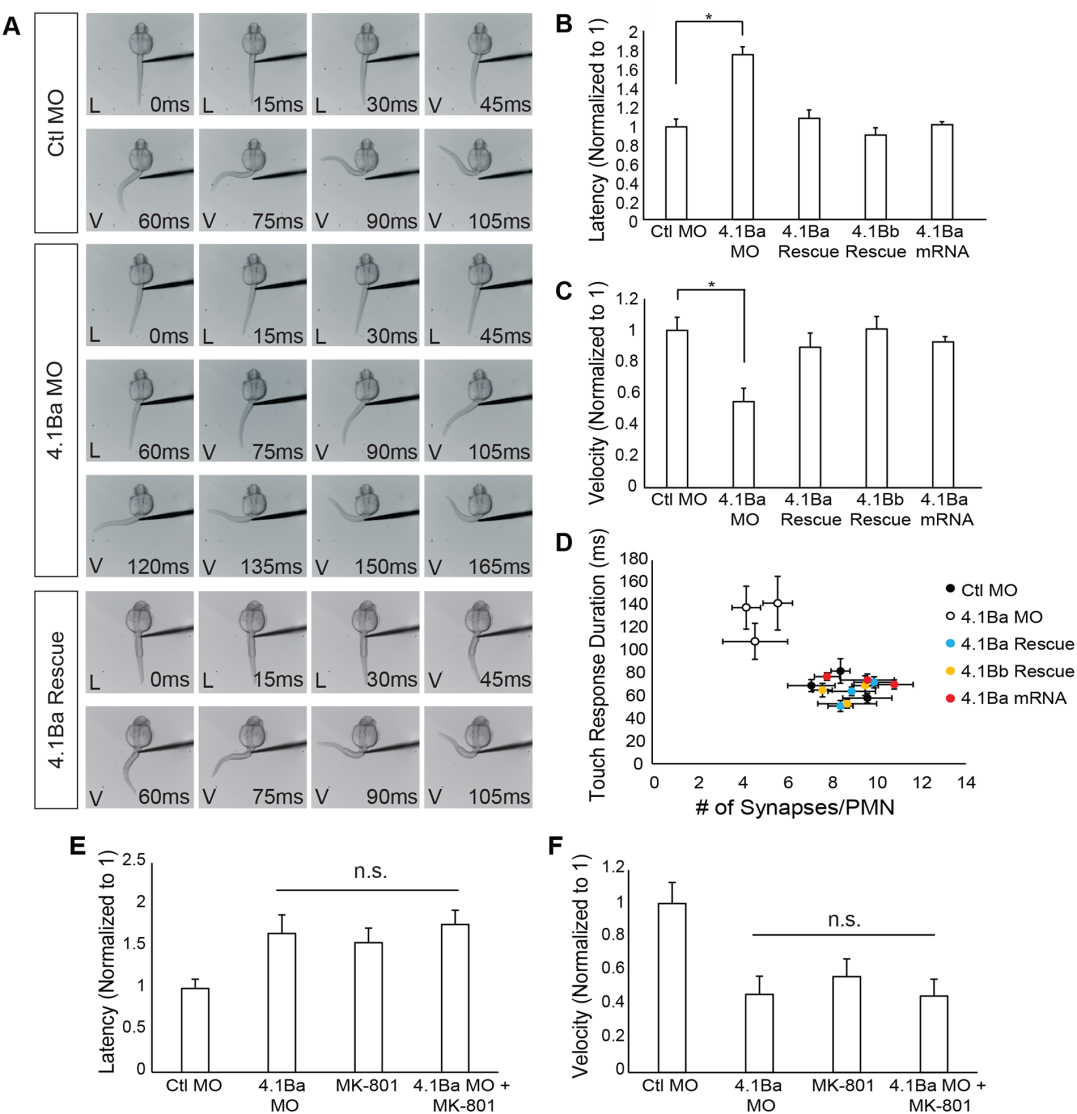
4.1Ba knockdown causes kinetic differences during touch-evoked coiling. (**A**) Example of touch-evoked coiling in Ctl MO, *4*.*1Ba* MO, and 4.1Ba Rescue embryos at 26 hpf. The time series depicts the full response used in our analysis to quantify the latency, L, and velocity, V, of C-bend responses. *4*.*1Ba* knockdown increased the latency (**B**) and decreased the velocity (**C**) of C-bend responses which was rescued with *4*.*1Ba* and *4*.*1Bb* RNA. (**D**) Correlation of touch-evoked response duration, defined as the latency plus time in motion, with synapse number for Ctl MO, *4*.*1Ba* MO, *4*.*1Ba* mRNA, and 4.1Ba and 4.1Bb rescues. (**E**) Latency and (**F**) velocity for touch-evoked coiling after inhibition of NMDA receptors with MK-801. No additive effect was seen when *4*.*1Ba* was knocked down and NMDA receptors were inhibited. N = 30 per condition for each experiment. Data represents three separate experiments normalized to 1 and evaluated with a one-way ANOVA followed by Tukey’s post-hoc analysis. Error bars represent s.e.m. * p < 0.05. Abbreviations: Ctl MO = control morpholino; 4.1Ba MO = *4*.*1Ba* morpholino; 4.1Ba Rescue = *4*.*1Ba* MO *+ 4*.*1Ba* mRNA; 4.1Bb Rescue = *4*.*1Ba* MO + *4*.*1Bb* mRNA.

We next performed a kinematic analysis of touch-evoked coiling with high-speed video recordings. The probability of stimulus-induced responses was unchanged, suggesting no sensory deficits (Part F in S2 Fig). In contrast, we found a significant increase in the latency (defined as the time it takes for the fish to begin to respond to a mechanical stimulus; 55% increase, p = 0.017; Fig 4A and 4B) and a significant decrease in the tail velocity (52% decrease, p = 0.017; Fig 4A and 4C) during single C-bend responses between Ctl MO and *4*.*1Ba* MO injected embryos. This phenotype was rescued by coinjection of *4*.*1Ba* and *4*.*1Bb* mRNAs (Fig 4A–4C). We labeled embryos after the touch tests were performed, and confirmed that the reduction in touch response duration, due to increased latency and decreased velocity of C-bends, correlated with a loss of synapse number at CaPs on an experiment by experiment basis (Fig 4D). This correlation strongly suggests the changes seen in touch-evoked coiling kinetics are due to an abnormal synaptic drive through CaPs. In contrast, *4*.*1Ba* misexpression did not alter the latency or the velocity of touch-evoked coiling (Fig 4B and 4C). Further, we found no differences in the number of spontaneous contractions per minute or velocity of C-bends during spontaneous contractions, which are mediated by electrical synapses (Parts G and H in S2 Fig). These results indicate 4.1Ba is exclusively required for chemical synapses used during touch-evoked coiling, and not for those involved in spontaneous motor behaviors.

The sensorimotor circuit is initially established with electrical synapses [24]. However, at 26 hpf, touch-evoked coiling becomes dependent upon glutamatergic signaling. Specifically, an inhibitor of AMPA-type glutamate receptors (CNQX) completely blocks the response, whereas inhibition of NMDA receptors with MK-801 results in a reduction in the overall duration of the touch response [20], similar to knock-down of *4*.*1Ba* (Fig 4A and 4B). Considering 4.1B is capable of enhancing the recruitment of NMDA receptors *in vitro* [16], we examined whether 4.1Ba might function together with NMDARs *in vivo*. Injection of embryos with the NMDA receptor inhibitor, MK-801, produced a 77% increase in latency and a 56% decrease in the velocity of C-bend responses. This result was similar to embryos injected with *4*.*1Ba* MO, which shows an 84% increase and 59% decrease in the latency and C-bend response, respectively (Latency: p = 0.014, Velocity: p < 0.001; Fig 4E and 4F). MK-801 injection into *4*.*1Ba* MO injected embryos did not enhance the effect. These experiments suggest 4.1Ba is necessary for establishing the synaptic drive underlying touch-evoked coiling, possibly through NMDA receptor-dependent mechanisms.

## Discussion

4.1B is a membrane-bound scaffolding molecule suggested to be involved in synapse formation *in vitro* [16]. To understand its role during synaptogenesis *in vivo*, we studied 4.1B in developing zebrafish embryos. We identified two *4*.*1B* gene orthologs in zebrafish, *4*.*1Ba*, expressed in the CNS, and *4*.*1Bb*, expressed in the brain and myotome. We identified 4.1Ba as important for synaptic integrity at PMNs, with trans-synaptic capabilities necessary for organizing the presynaptic specialization. We also found a loss of *4*.*1Ba* altered touch-evoked coiling at 26 hpf after MO knockdown, which correlated with loss of synapses at PMNs. Together, these data confirm 4.1B’s role in glutamatergic synapse formation and establishes its importance in mediating fine control of touch-evoked coiling. These data also highlight the use of zebrafish for studying 4.1 proteins during synaptogenesis.

### The zebrafish genome contains two *4*.*1B* orthologs

We identified two *4*.*1B* orthologs in the zebrafish genome, confirmed by synteny and phylogenetic analysis (Fig 1A and 1E). Individual domains of these proteins and the underlying genetic code show great homology with their mammalian counterpart (Fig 1B–1D) with minor differences. Interestingly, only *4*.*1Ba* contained the SAB domain. The SAB domain evolved in higher organisms and is thought to be a defining feature of 4.1 function (reviewed in [4]). This loss of function in *4*.*1Bb* suggests the encoded proteins have subfunctionalized their activities.

Further, *in situ* hybridization is consistent with the hypothesis of subfunctionalization. *4*.*1Ba* and *4*.*1Bb* are expressed in the telencephalon, mesencephalon, and retina, and may play redundant or compensatory roles in these tissues. In the trunk, only *4*.*1Ba* was expressed in the spinal cord, including in CaP PMNs (Fig 2E) and CoPA interneurons (Fig 2F), whereas *4*.*1Bb* was expressed exclusively in the myotome (Fig 2G). The divergence of expression patterns in the trunk suggests each gene may have acquired subtle differences in function based on the needs of the tissue they are expressed in, with 4.1Ba important for establishing the sensorimotor circuit at early developmental stages.

### 4.1Ba is necessary for the formation of synapses

By 26 hpf, zebrafish have developed the sensorimotor circuit, a reflex pathway that allows zebrafish to respond to a tactile stimulus. The cells of this pathway are restricted to the spinal cord and are well characterized [20, 24], allowing us to study the functional consequences of *4*.*1Ba* knockdown on synapses and behavior. Knockdown of *4*.*1Ba* led to a decrease in excitatory pre- and postsynaptic specializations, and synapse number at CaPs (Fig 3), but no change was observed at CoPA cells (Parts D and E in S1 Fig). Observing different results in CoPAs vs CaPs was surprising as our method of knockdown was global and not targeted. Loss of *4*.*1Ba* causing an effect only at CaP synapses and not at CoPA synapses suggests 4.1Ba may serve different functions dependent on the cell type it is expressed in. No effect was seen on the number of inhibitory or cholinergic synapses found at CaPs (Parts H and I in S1 Fig, and Part A in S2 Fig), providing evidence that 4.1Ba is important exclusively for the stabilization of glutamatergic synapses at CaPs.

Since loss of *4*.*1Ba* caused a reduction in the number of presynaptic puncta at CaPs, we determined whether this was a cell autonomous, or non-cell autonomous effect (Fig 3F). *4*.*1Ba* is expressed throughout the spinal cord, and it is therefore possible presynaptic 4.1Ba is responsible for the recruitment of presynaptic components. We found the number of synapsin puncta, and overall synapse number, was rescued in CaPs expressing 4.1Ba-GFP when coinjected with the *4*.*1Ba* MO. This result supports the hypothesis that 4.1Ba does not act within the presynaptic terminal, but plays a trans-synaptic role in stabilizing the presynaptic terminal. This might be through a tight interaction with cell adhesion molecules, such as Synaptic Cell Adhesion Molecules (SynCAMs) [16], which were recently shown to also drive presynaptic assembly through a postsynaptic mechanism [30].

### 4.1Ba is important for the function of the sensorimotor circuit

The loss of synapses at PMN cell bodies suggests neuronal signaling might be altered. To study the functional consequences of the loss of 4.1Ba, we examined touch-evoked coiling dependent on CaPs. We observed a significant increase in the time it takes the zebrafish to respond to the mechanical stimulus, as well as a significant decrease in the velocity of the C-bend response (Fig 4A–4C). The behavioral changes observed correlated with a loss of synapse number (Fig 4D). We found no abnormalities in the muscle nor did we observe any changes in the gross structure of NMJs, providing further support that the phenotypes observed were due to loss of synapses at CaPs. We also found no changes in the frequency or velocity of spontaneous responses, indicating 4.1Ba is only involved in chemical synapse formation (Parts G and H in S2 Fig). It is important to note that 4.1B is important for organizing the myelin sheath around axons, but at the age examined in this study, myelin has not yet formed around CaP axons [31].

Finally, we examined the role of 4.1Ba during touch-evoked behaviors in the presence of an inhibitor to NMDA receptors. Previous studies have shown the NMDA receptor antagonist MK-801 does not affect spontaneous responses but does increase the duration of touch-evoked behaviors [20], similar to the results obtained with *4*.*1Ba* knockdown. We found no difference in the velocity or latency of touch-evoked responses when *4*.*1Ba* was knocked-down and NMDA receptors were inhibited. These data suggest these two proteins may function within the same pathway, though further studies are needed to determine if a direct link exists between 4.1Ba and the NMDA receptor. These data further support the importance of 4.1Ba at glutamatergic synapses and their involvement in touch-evoked motor control.

In summary, we identified 4.1Ba as an important component of excitatory synapse formation. The loss of *4*.*1Ba* caused a reduction in the number of synapses on CaPs, providing less glutamatergic transmission to mediate touch-evoked coiling. This work confirms previous *in vitro* data that suggest 4.1B is important for glutamatergic synapse formation, and establishes the functional importance of 4.1B at synapses *in vivo* within the sensorimotor circuit.

## Materials and methods

### Vertebrate animals

Experiments using zebrafish were carried out in strict accordance with the recommendations in the Guide for the Care and Use of Laboratory Animals of the National Research Council. The protocols were approved by the University of Oregon Institutional Animal Care and Use Committee (Permit Number: #13–21). All zebrafish were of the AB/Tübingen, tg(*mnx1*:*Gal4;UAS*:*GFP*), or tg(*mnx1*:*Gal4*) line [23], and were raised and maintained in the University of Oregon Zebrafish Facility at 28.5°C with a 14/10 light/dark cycle according to standard protocols [32]. Zebrafish embryos were anesthetized with 0.003% MS-222 (Western Chemical, Ferndale, WA) prior to fixation or embedding in 1.5% low melt agarose in embryo medium for live imaging.

### Identification of *4*.*1B* genes

To obtain transcripts of the zebrafish *4*.*1B* genes, predicted sequences were identified from the zebrafish genome assembly Zv9 from the Sanger Institute (http://uswest.ensembl.org/Danio_rerio/Info/Index) using mouse *epb41l3* (*4*.*1B*) mRNA sequence for comparison (NM_013813.1). This search revealed two *4*.*1B* genes, named *epb41l3a* (*4*.*1Ba*—Gene ID 445478) and *epb41l3b* (*4*.*1Bb*—Gene ID 407981) which were examined by synteny analysis (http://teleost.cs.uoregon.edu/acos/synteny_db/) [33]. Deduced mRNA and protein sequences were aligned to 4.1B, 4.1N, 4.1G, and 4.1R from various organisms using ClustalW to predict nucleic acid and amino acid sequence homology of individual exons and domains respectively [34]. To generate the phylogenetic tree, sequences were trimmed to include unambiguously aligned regions and were analyzed by Phylogeny.fr [22]. Image clones of both *4*.*1B* genes were obtained (GE Dharmacon, Lafayette, CO; *4*.*1Ba*, Clone ID: 7043476; *4*.*1Bb*: Clone ID: 6967899) and cloned into pXT7 for capped mRNA synthesis by PCR-amplification with primers incorporating EcoRI and XhoI restriction sites in the 5’ and 3’ primers respectively. mRNA was synthesized with the mMESSAGE mMACHINE T7 transcription kit (Life Technologies, Grand Island, NY).

### Reverse transcriptase PCR

RNA was isolated from AB/Tübingen embryos at the 16-cell, 90% epiboly, 3-somite, 16, 24, 48, 72 hpf, and 7 dpf stages using Trizol Reagent (Invitrogen, Carlsbad, CA). cDNA was synthesized with the SuperScript III First-Strand Synthesis system (Invitrogen, Carlsbad, CA) and concentrations of the cDNAs were determined and adjusted against the alpha-tubulin gene. Primers were designed to unique regions of *4*.*1Ba* and *4*.*1Bb* for PCR amplification (S1 Table). PCR products for 16-cell, 90% epiboly, 24 hpf, and 7 dpf embryos were confirmed for specificity by sequencing.

### *In situ* hybridization

Zebrafish embryos, treated with 0.003% 1-phenyl-2-thiourea to prevent pigmentation, were staged according to Kimmel et al. (1995) at 26 hpf [35]. Sense and antisense probes were synthesized from cloned cDNAs with primers that contained T7 or T3 promoter sites (S2 Table). Probes were designed to unique regions of the *4*.*1B* genes, and were confirmed for specificity by BLAST analysis. The probes were subsequently tagged with digoxigenin, and whole-mount *in situ* hybridization was performed as previously described [36]. Sections were cut at a thickness of 16 μm. Images were acquired on a Leica M165 FC stereomicroscope using a Leica DFC425 C digital camera and processed in Adobe Photoshop. FISH was carried out as previously described [37]. Sense probes for the *mafba* gene were synthesized as above, and subsequently tagged with dinitrophenyl to label CoPA cells [26]. Following FISH, embryos were incubated with either rabbit anti-GFP (1:500; Life Technologies, Grand Island, NY) or chicken anti-GFP antibodies (1:500; Abcam, Cambridge, MA) and labeled with anti-rabbit-Alexa488 or anti-chicken-Alexa488 (Life Technologies, Grand Island, NY) for visualization. We used faint background labeling of the ck-GFP antibody to identify the muscle following FISH. Images were acquired on an Olympus FV1000 confocal microscope, and intensity of *4*.*1Ba* mRNA expression in segments 8–12 was quantified using ImageJ. Intensity was calculated for mRNA in CaPs (5 per image) and mRNA in CoPAs (2–3 per image) and compared to total mRNA. The experiments were repeated 3 times, with N ≥ 10 embryos per condition.

### *4*.*1Ba* and *4*.*1Bb* morpholino knockdown

Antisense morpholino oligonucleotides (MO; Gene Tools, Philomath, OR; S3 Table) were designed to unique target splice sites in each gene and 2 nL was injected into embryos at the one cell stage using an MPPI-2 pressure injector (ASI, Eugene, OR). The appropriate concentration of the MOs was determined by examining a concentration gradient from 0.1mM to 1.2mM. A concentration of 0.8 mM produced the strongest specific phenotype, without adverse effects.

We determined the specificity of action of the MOs using the following controls: (1) We confirmed knockdown in every experiment by performing RT-PCR from 26 hpf embryos using primers to exonic sequences flanking the targeted splice site (S4 Table). In both cases, RT-PCR products were larger by the expected size of intron 1 in MO injected animals (Parts A and B in S1 Fig), and the RT-PCR products were confirmed for inclusion of intron 1 by sequencing [28]. (2) We examined whether MOs had any adverse effects on overall embryonic development, by visual inspection of embryos and by assessing cell death with acridine orange [38] (Part C in S1 Fig). No effect on morphology or cell death was detected. (3) We compared all knockdowns to embryos injected with a scrambled Ctl MO. (4) We performed rescue and misexpression experiments of MO-mediated knockdown by co-injecting *in vitro* transcribed mRNA encoding zebrafish image clones of *4*.*1Ba* or *4*.*1Bb* in pXT7 at 20 ng μL^-1^ (Fig 3B–3E). Appropriate mRNA concentrations were determined by injecting a gradient of mRNA from 5 ng μL^-1^ to 50 ng μL^-1^. Concentrations above 25 ng μL^-1^ were toxic. (5) We performed a cell-specific rescue by injecting a plasmid encoding *UAS*:*4*.*1Ba-GFP* into the tg(*mnx1*:*Gal4*) line. (Fig 3F, and see ‘Cell-specific rescue of 4.1Ba in CaPs’ below). We concluded from these extensive controls that the MOs do not have off-target effects at the concentrations used.

### Cell-specific rescue of 4.1Ba in CaPs

Fertilized eggs derived from a cross of tg(*mnx1*:*Gal4;UAS*:*GFP*) and tg(*mnx1*:*Gal4*) zebrafish were injected with the Ctl MO, *4*.*1Ba* MO, or the *4*.*1Ba* MO plus a DNA plasmid encoding *UAS*:*4*.*1Ba-GFP*. The DNA was injected at 20ng uL^-1^ with pCS2FA-transposase mRNA at 100ng μL^-1^. Appropriate concentrations were determined by injecting tg(*mnx1*:*Gal4*) embryos with combinations of escalating concentrations of *UAS*:*4*.*1Ba-GFP* and transposase. The reported concentration of both constructs produced the highest number of embryos expressing 4.1Ba-GFP in CaPs. For the rescue, embryos expressing only 4.1Ba GFP were chosen based on their distinct pattern of GFP expression (Fig 3A) in a subset of MNs, and were compared to embryos expressing GFP in all primary and secondary MNs [23]. CaPs were chosen for analysis based on their long descending axon along the ventral myotome, with no visible axons from other neurons running close to the cell body. This ensured any molecular phenotypes observed were due to postsynaptic 4.1B function and not presynaptic 4.1B. For embryos injected with the Ctl MO or *4*.*1Ba* MO only, CaPs from embryos expressing GFP in all MNs were chosen for study. Three CaPs were selected per embryo for analysis. Embryos were anesthetized at 26 hpf in 0.003% MS-222, fixed with 4% paraformaldehyde and processed for immunolabeling as described below.

### Immunolabeling

Immunofluorescence labeling was performed as previously described [18]. Embryos were staged at 26 hpf and fixed for either 1.5 hrs at 4°C (panMAGUK, Synapsin 1/2, GFP), for 3 hrs at 4°C (Gephyrin, Phalloidin, α-Bungarotoxin, GFP) or overnight at 4°C (SV2, Synaptotagmin 2b, GFP) in 1X Fish Fix Buffer [32] and 4% paraformaldehyde. Samples were rinsed in PBS with 0.1% Triton X-100 (PBST) after fixation, and then blocked in PBS with 1% BSA, 1% DMSO, 2% Normal Goat Serum, and 0.1% Triton-X 100. Embryos were then incubated with the primary antibodies overnight, washed in PBST, and incubated with the secondary antibodies for 5 hours at room temperature. Samples were washed again in PBST and stored in 80% glycerol. The following primary antibodies and dilutions were used: mouse anti-panMAGUK (1:100; NeuroMab, Davis, CA) [18, 27], rabbit anti-Synapsin 1/2 (1:1000; Synaptic Systems, Goettingen, Germany) [18], chicken anti-GFP (1:500; Abcam, Cambridge, MA), mouse anti-Synaptotagmin2b (znp-1; 1:750; ZIRC, Eugene, OR) [18], mouse anti-SV2 (1:1000; Developmental Studies Hybridoma Bank, University of Iowa) [18], mouse anti-Gephyrin (1:500; Synaptic Systems, Goettingen, Germany) [39], Tetramethylrhodamine α-Bungarotoxin (1:500; Life Technologies, Grand Island, NY), and Alexa488 Phalloidin (1:20; Life Technologies, Grand Island, NY). Primary antibodies were evaluated against published studies and were confirmed to produce the same labeling patterns as previously described. Secondary antibodies used were anti-Chicken Alexa488, anti-mouse Alexa546, and anti-rabbit Alexa633 (Molecular Probes, Eugene, OR). Images were taken of the spinal cord from somites 8–12 on an Olympus Fluoview FV1000 confocal microscope with a 63x oil-immersion objective. Images were processed into a flattened RGB composite as a maximum intensity projection and saved as tiff files in ImageJ version 1.43 with the loci tools plugin. These images were analyzed using the puncta analyzer plugin [40] in ImageJ version 1.29. Three CaPs (n) were analyzed per embryo (N) and the total number of puncta per CaP was averaged [40]. All experiments were repeated at least 3 times, with N ≥ 10 embryos per condition. Fish were excluded if no identifiable CaPs were visible. CaPs were chosen based on their long projecting axon down the ventral myotome [25]. Neuronal morphology was analyzed with BONFIRE [41]. Data was analyzed twice by two separate experimenters, the second of which was blinded to conditions. All data was analyzed in SPSS with a two-tailed unpaired student t-test, or a one way-ANOVA followed by Tukey’s *post hoc* analysis; p < 0.05 was considered to be significant. All results represent an individual experiment, and error bars represent standard error of the mean (s.e.m).

### Video recording and kinematic analysis

Analysis of embryo behavior was performed as previously described [20]. Briefly, embryos were anesthetized in 0.003% MS-222 and mounted in 1.5% low melt agarose in embryo medium (EM). The embryos were then submerged in EM without MS-222 and the agarose surrounding the tail was removed. The movements of embryos were recorded under a stereomicroscope (Leica LZMFIII) using a Phantom v4.2 camera at a rate of 300 frames per second. We recorded 20 hpf embryos for 1 minute and counted the frequency of tail swings and velocity of spontaneous contractions using Image Pro Plus software (Media Cybernetics, Bethesda, MD) of the first five tail swings of each embryo. Touch-evoked coiling was measured at 26 hpf by stimulating embryos with an insect pin attached to a micromanipulator five times. Latency was measured from the time the mechanical stimulus touched the skin until voluntary muscle contraction was observed. Velocity was analyzed by measuring the distance and time beginning at the tip of the tail prior to voluntary contraction, to the tip of the tail at max deflection for all five tail swings. This was averaged to give a single velocity for each individual embryo. For inhibition of NMDA receptors, embryos were injected at 24 hpf with either 8 nL of 100 μM MK-801 or 8 nL of EM into the hindbrain [20, 32]. Embryos were allowed to recover for 1 hour, then touch-evoked responses were acquired and analyzed as described above. Experiments were repeated three times with a minimum of 10 fish per condition and normalized to 1 for comparing results across experiments. Statistical analysis including two-tailed unpaired student t-tests and one-way ANOVA’s, followed by Tukey’s *post hoc* analysis, were carried out in SPSS. Variance between groups was similar and data was normally distributed. p < 0.05 was considered to be significant. All results are expressed as s.e.m.

## Supporting information

S1 Fig 4.1Ba MO specifically affects glutamatergic synapses at CaPs. (**A**) Schematic of 4.1Ba knockdown. The 4.1Ba MO (blue box) is designed between exon 1 (E1) and intron 1 to incorporate intron 1 in the mRNA. Intron 1 contains an early stop codon (purple line), leading to a truncated, nonfunctional protein. Primers designed in E1 (green arrow) and E2 (red arrow) were used for RT-PCR and sequencing products. (**B**) Confirmation of 4.1Ba knockdown by RT-PCR. RT-PCR products were larger by the expected size of intron 1 in MO injected animals. The 4.1Ba MO caused an 86% reduction in properly spliced transcripts that were confirmed by sequencing. (**C**) Acridine Orange staining demonstrates no difference in cell death between Ctl MO and 41Ba MO. Red box represents the spinal cord region examined. Dotted lines represent the edges of the spinal cord (SC). (**D**) Representation and (**E**) quantification of Syn and PM labeling at CoPA cells. No changes in the number of pre- or postsynaptic markers were found. (**F**) Representation and (**G**) quantification of Syn and PM labeling after knockdown of both 4.1B proteins, 4.1Ba and 4.1Bb alone, and misexpression of 4.1Bb. 4.1Bb knockdown did not affect synapse number, nor did it enhance the phenotype associated with 4.1Ba knockdown. No effect was seen with 4.1Bb misexpression. (**H**) Representation and (**I**) quantification of Gephyrin labeling at CaPs. Loss of 4.1Ba did not affect the number of inhibitory synapses at CaPs. N = 10 for all conditions and experiments. Significance was evaluated with a One-way ANOVA followed by Tukey’s post-hoc analysis. All experiments were repeated in triplicate. Error bars represent s.e.m. * p < 0.05; ** p< 0.01. Scale bar in B, D: 5 μm. Abbreviations: Ctl MO = control morpholino; 4.1Ba MO = 4.1Ba morpholino; 4.1Ba Rescue = 4.1Ba MO + 4.1Ba mRNA; 4.1Bb MO = 4.1Bb morpholino.
(PDF)

S2 Fig 4.1Ba MO does not alter structures involved in the sensorimotor circuit. (**A**) Lateral view of CaPs, the myotome, and the NMJ in the trunk of 26 hpf Ctl MO and 4.1Ba MO injected embryos. (**B—D**) No differences were found in the number of branch points and terminal points (**B**), number of processes per cell (**C**), or the length of the processes **(D**). (**E**) Quantification of acetylcholine puncta at the neuromuscular junction. There were no changes in the number of acetylcholine puncta at the NMJ. (**F**) Failure rate of touch-evoked responses represented as a percentage of stimuli. No effect was seen in the ability of the embryos to respond to a tactile stimulus. (**G**) Frequency of spontaneous coils represented as the number of spontaneous coils per minute at 19 hpf. (**H**) Kinematic analysis of individual C-tail bends at 19 hpf. No differences were seen in the frequency or velocity of spontaneous coils. Error bars: s.e.m. Scale bar in A: 10 μm—CaPs; 50 μm—muscle and NMJ.
(PDF)

S1 Table Primers used for RT-PCR. (PDF)

S2 Table Primers used for *in situ* hybridization. (PDF)

S3 Table MO nucleotide sequences. (PDF)

S4 Table Primers used to confirm MO knockdown. (PDF)

## References

[1] Ryan TJ, Grant SG. The origin and evolution of synapses. Nat Rev Neurosci. 2009;10(10):701–12. 10.1038/nrn2717 19738623

[2] Yin J, Schaaf CP. Autism genetics—an overview. Prenat Diagn. 2016. 10.1002/pd.4942 27743394

[3] Escudero I, Johnstone M. Genetics of schizophrenia. Curr Psychiatry Rep. 2014;16(11):502 10.1007/s11920-014-0502-8 25200985 PMC6192508

[4] Baines AJ, Lu HC, Bennett PM. The Protein 4.1 family: hub proteins in animals for organizing membrane proteins. Biochim Biophys Acta. 2013;1838(2):605–19. 10.1016/j.bbamem.2013.05.030 23747363

[5] Parra M, Gascard P, Walensky LD, Gimm JA, Blackshaw S, Chan N, et al Molecular and functional characterization of protein 4.1B, a novel member of the protein 4.1 family with high level, focal expression in brain. J Biol Chem. 2000;275(5):3247–55. 10652311 10.1074/jbc.275.5.3247

[6] Scott C, Keating L, Bellamy M, Baines AJ. Protein 4.1 in forebrain postsynaptic density preparations: enrichment of 4.1 gene products and detection of 4.1R binding proteins. Eur J Biochem. 2001;268(4):1084–94. 11179975 10.1046/j.1432-1327.2001.01968.x

[7] Gimm JA, An X, Nunomura W, Mohandas N. Functional characterization of spectrin-actin-binding domains in 4.1 family of proteins. Biochemistry. 2002;41(23):7275–82. 12044158 10.1021/bi0256330

[8] Scott C, Phillips GW, Baines AJ. Properties of the C-terminal domain of 4.1 proteins. Eur J Biochem. 2001;268(13):3709–17. 11432737 10.1046/j.1432-1327.2001.02276.x

[9] Sun CX, Robb VA, Gutmann DH. Protein 4.1 tumor suppressors: getting a FERM grip on growth regulation. J Cell Sci. 2002;115(Pt 21):3991–4000. 12356905 10.1242/jcs.00094

[10] Baines AJ. A FERM-adjacent (FA) region defines a subset of the 4.1 superfamily and is a potential regulator of FERM domain function. BMC Genomics. 2006;7:85 10.1186/1471-2164-7-85 16626485 PMC1459144

[11] Yageta M, Kuramochi M, Masuda M, Fukami T, Fukuhara H, Maruyama T, et al Direct association of TSLC1 and DAL-1, two distinct tumor suppressor proteins in lung cancer. Cancer Res. 2002;62(18):5129–33. 12234973

[12] Terada N, Ohno N, Saitoh S, Seki G, Komada M, Suzuki T, et al Interaction of membrane skeletal protein, protein 4.1B and p55, and sodium bicarbonate cotransporter1 in mouse renal S1-S2 proximal tubules. J Histochem Cytochem. 2007;55(12):1199–206. 10.1369/jhc.7A7266.2007 17712176

[13] An X, Zhang X, Debnath G, Baines AJ, Mohandas N. Phosphatidylinositol-4,5-biphosphate (PIP2) differentially regulates the interaction of human erythrocyte protein 4.1 (4.1R) with membrane proteins. Biochemistry. 2006;45(18):5725–32. 10.1021/bi060015v 16669616

[14] Baines AJ. Evolution of spectrin function in cytoskeletal and membrane networks. Biochem Soc Trans. 2009;37(Pt 4):796–803. 10.1042/BST0370796 19614597

[15] Shen L, Liang F, Walensky LD, Huganir RL. Regulation of AMPA receptor GluR1 subunit surface expression by a 4. 1N-linked actin cytoskeletal association. J Neurosci. 2000;20(21):7932–40. 11050113 10.1523/JNEUROSCI.20-21-07932.2000 PMC6772741

[16] Hoy JL, Constable JR, Vicini S, Fu Z, Washbourne P. SynCAM1 recruits NMDA receptors via protein 4.1B. Mol Cell Neurosci. 2009;42(4):466–83. 10.1016/j.mcn.2009.09.010 19796685 PMC2784006

[17] Wozny C, Breustedt J, Wolk F, Varoqueaux F, Boretius S, Zivkovic AR, et al The function of glutamatergic synapses is not perturbed by severe knockdown of 4.1N and 4.1G expression. J Cell Sci. 2009;122(Pt 5):735–44. 10.1242/jcs.037382 19225127

[18] Easley-Neal C, Fierro J Jr, Buchanan J, Washbourne P. Late recruitment of synapsin to nascent synapses is regulated by Cdk5. Cell Rep. 2013;3(4):1199–212. 10.1016/j.celrep.2013.03.031 23602570 PMC3742072

[19] Saint-Amant L, Drapeau P. Synchronization of an embryonic network of identified spinal interneurons solely by electrical coupling. Neuron. 2001;31(6):1035–46. 11580902 10.1016/s0896-6273(01)00416-0

[20] Pietri T, Manalo E, Ryan J, Saint-Amant L, Washbourne P. Glutamate drives the touch response through a rostral loop in the spinal cord of zebrafish embryos. Dev Neurobiol. 2009;69(12):780–95. 10.1002/dneu.20741 19634126 PMC2771646

[21] Postlethwait J, Ruotti V, Carvan MJ, Tonellato PJ. Automated analysis of conserved syntenies for the zebrafish genome. Methods Cell Biol. 2004;77:255–71. 15602916 10.1016/s0091-679x(04)77014-4

[22] Dereeper A, Guignon V, Blanc G, Audic S, Buffet S, Chevenet F, et al Phylogeny.fr: robust phylogenetic analysis for the non-specialist. Nucleic Acids Res. 2008;36(Web Server issue):W465–9. 10.1093/nar/gkn180 18424797 PMC2447785

[23] Zelenchuk TA, Bruses JL. In vivo labeling of zebrafish motor neurons using an mnx1 enhancer and Gal4/UAS. Genesis. 2011;49(7):546–54. 10.1002/dvg.20766 21538811 PMC3642388

[24] Saint-Amant L, Drapeau P. Time course of the development of motor behaviors in the zebrafish embryo. J Neurobiol. 1998;37(4):622–32. 9858263 10.1002/(sici)1097-4695(199812)37:4<622::aid-neu10>3.0.co;2-s

[25] Westerfield M, McMurray JV, Eisen JS. Identified motoneurons and their innervation of axial muscles in the zebrafish. J Neurosci. 1986;6(8):2267–77. 3746409 10.1523/JNEUROSCI.06-08-02267.1986 PMC6568761

[26] Wells S, Nornes S, Lardelli M. Transgenic zebrafish recapitulating tbx16 gene early developmental expression. PLoS One. 2011;6(6):e21559 10.1371/journal.pone.0021559 21720556 PMC3123366

[27] Meyer MP, Trimmer JS, Gilthorpe JD, Smith SJ. Characterization of zebrafish PSD-95 gene family members. J Neurobiol. 2005;63(2):91–105. 10.1002/neu.20118 15660367

[28] Eisen JS, Smith JC. Controlling morpholino experiments: don’t stop making antisense. Development. 2008;135(10):1735–43. 10.1242/dev.001115 18403413

[29] Kneussel M, Betz H. Receptors, gephyrin and gephyrin-associated proteins: novel insights into the assembly of inhibitory postsynaptic membrane specializations. J Physiol. 2000;525 Pt 1:1–9. 10811719 10.1111/j.1469-7793.2000.t01-4-00001.x PMC2269938

[30] Fowler DK, Peters JH, Williams C, Washbourne P. Redundant Postsynaptic Functions of SynCAMs 1–3 during Synapse Formation. Front Mol Neurosci. 2017;10:24 10.3389/fnmol.2017.00024 28197078 PMC5281628

[31] Brosamle C, Halpern ME. Characterization of myelination in the developing zebrafish. Glia. 2002;39(1):47–57. 10.1002/glia.10088 12112375

[32] Westerfield M. The Zebrafish Book, Fourth Edition (Eugene, OR: Institute for Neuroscience, University of Oregon). 2000.

[33] Catchen JM, Conery JS, Postlethwait JH. Automated identification of conserved synteny after whole-genome duplication. Genome Res. 2009;19(8):1497–505. 10.1101/gr.090480.108 19465509 PMC2720179

[34] Subramaniam S. The Biology Workbench—a seamless database and analysis environment for the biologist. Proteins. 1998;32(1):1–2. 9672036

[35] Kimmel CB, Ballard WW, Kimmel SR, Ullmann B, Schilling TF. Stages of embryonic development of the zebrafish. Dev Dyn. 1995;203(3):253–310. 10.1002/aja.1002030302 8589427

[36] Hauptmann G, Gerster T. Multicolor whole-mount in situ hybridization. Methods Mol Biol. 2000;137:139–48. 10.1385/1-59259-066-7:139 10948532

[37] Welten MC, de Haan SB, van den Boogert N, Noordermeer JN, Lamers GE, Spaink HP, et al ZebraFISH: fluorescent in situ hybridization protocol and three-dimensional imaging of gene expression patterns. Zebrafish. 2006;3(4):465–76. 10.1089/zeb.2006.3.465 18377226

[38] Williams JA, Holder N. Cell turnover in neuromasts of zebrafish larvae. Hear Res. 2000;143(1–2):171–81. 10771194 10.1016/s0378-5955(00)00039-3

[39] Schneider Gasser EM, Straub CJ, Panzanelli P, Weinmann O, Sassoe-Pognetto M, Fritschy JM. Immunofluorescence in brain sections: simultaneous detection of presynaptic and postsynaptic proteins in identified neurons. Nat Protoc. 2006;1(4):1887–97. 10.1038/nprot.2006.265 17487173

[40] Ippolito DM, Eroglu C. Quantifying synapses: an immunocytochemistry-based assay to quantify synapse number. J Vis Exp. 2010(45). 10.3791/2270 PMC3159596 21113117

[41] Kutzing MK, Langhammer CG, Luo V, Lakdawala H, Firestein BL. Automated Sholl analysis of digitized neuronal morphology at multiple scales. J Vis Exp. 2010(45). 10.3791/2354 PMC3159598 21113115

