# Supplementary figures and images for: 4.1Ba is necessary for glutamatergic synapse formation in the sensorimotor circuit of developing zebrafish
Source: PLoS One. 2018 Oct 4;13(10):e0205255. doi: 10.1371/journal.pone.0205255 (PMC6171929; doi:10.1371/journal.pone.0205255)

**S1 Fig**

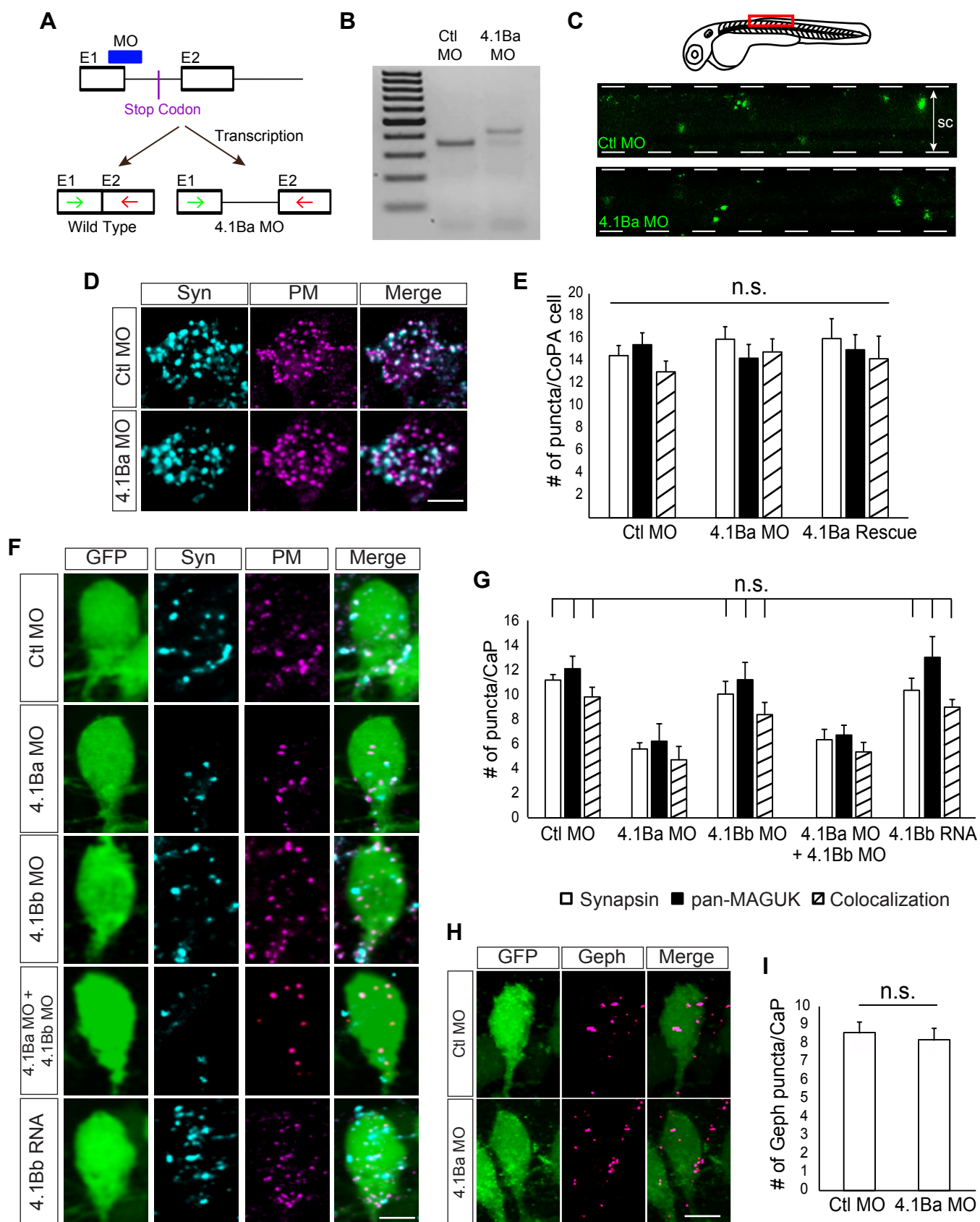

Supplement: S1 Fig — (A) Schematic of 4.1Ba knockdown. The 4.1Ba MO (blue box) is designed between exon 1 (E1) and intron 1 to incorporate intron 1 in the mRNA. Intron 1 contains an early stop codon (purple line), leading to a truncated, nonfunctional protein. Primers designed in E1 (green arrow) and E2 (red arrow) were used for RT-PCR and sequencing products. (B) Confirmation of 4.1Ba knockdown by RT-PCR. RT-PCR products were larger by the expected size of intron 1 in MO injected animals. The 4.1Ba MO caused an 86% reduction in properly spliced transcripts that were confirmed by sequencing. (C) Acridine Orange staining demonstrates no difference in cell death between Ctl MO and 41Ba MO. Red box represents the spinal cord region examined. Dotted lines represent the edges of the spinal cord (SC). (D) Representation and (E) quantification of Syn and PM labeling at CoPA cells. No changes in the number of pre- or postsynaptic markers were found. (F) Representation and (G) quantification of Syn and PM labeling after knockdown of both 4.1B proteins, 4.1Ba and 4.1Bb alone, and misexpression of 4.1Bb. 4.1Bb knockdown did not affect synapse number, nor did it enhance the phenotype associated with 4.1Ba knockdown. No effect was seen with 4.1Bb misexpression. (H) Representation and (I) quantification of Gephyrin labeling at CaPs. Loss of 4.1Ba did not affect the number of inhibitory synapses at CaPs. N = 10 for all conditions and experiments. Significance was evaluated with a One-way ANOVA followed by Tukey’s post-hoc analysis. All experiments were repeated in triplicate. Error bars represent s.e.m. * p < 0.05; ** p< 0.01. Scale bar in B, D: 5 μm. Abbreviations: Ctl MO = control morpholino; 4.1Ba MO = 4.1Ba morpholino; 4.1Ba Rescue = 4.1Ba MO + 4.1Ba mRNA; 4.1Bb MO = 4.1Bb morpholino. (PDF) [file pone.0205255.s001.pdf]

S2 Fig

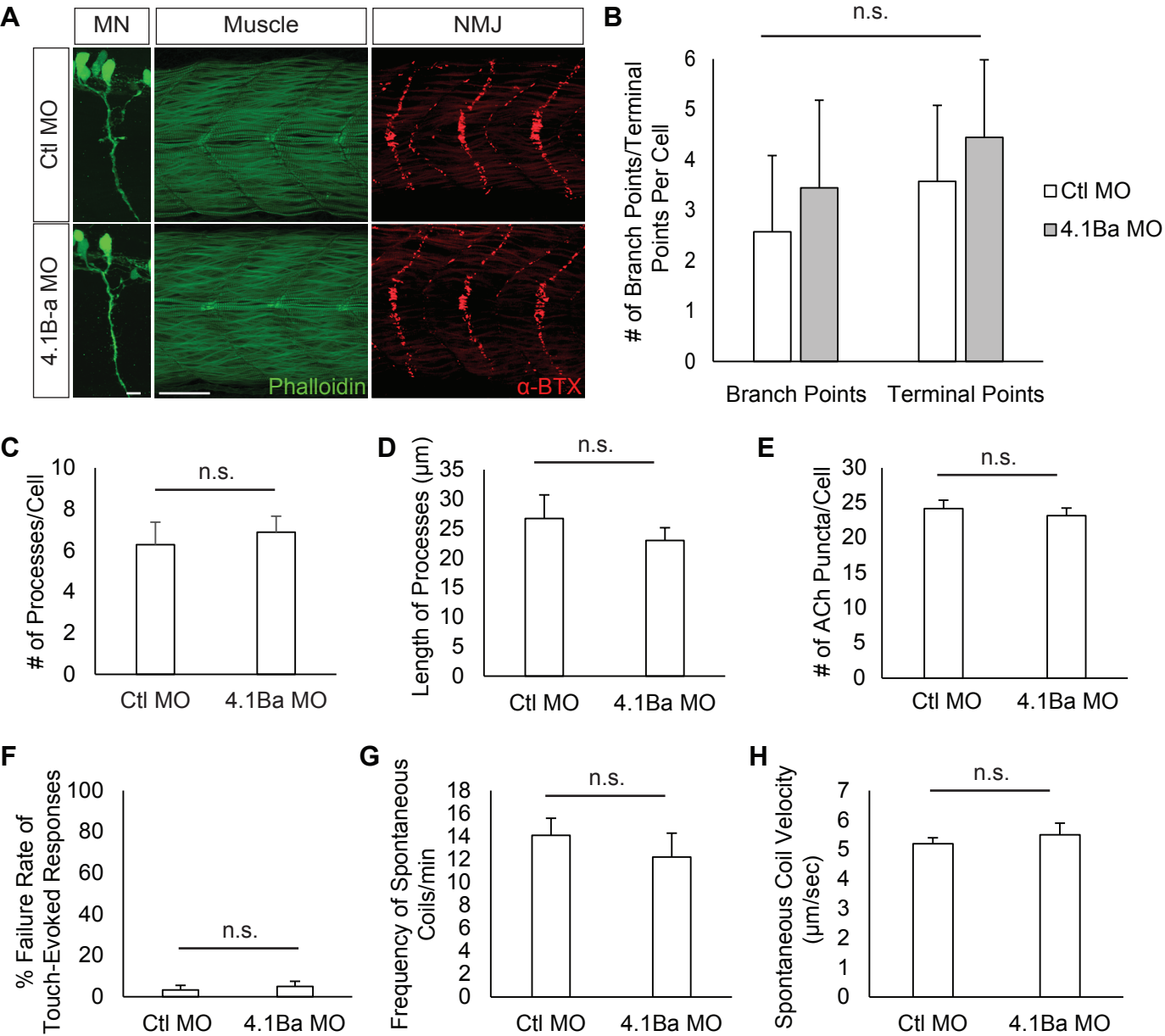

Supplement: S2 Fig — (A) Lateral view of CaPs, the myotome, and the NMJ in the trunk of 26 hpf Ctl MO and 4.1Ba MO injected embryos. (B—D) No differences were found in the number of branch points and terminal points (B), number of processes per cell (C), or the length of the processes (D). (E) Quantification of acetylcholine puncta at the neuromuscular junction. There were no changes in the number of acetylcholine puncta at the NMJ. (F) Failure rate of touch-evoked responses represented as a percentage of stimuli. No effect was seen in the ability of the embryos to respond to a tactile stimulus. (G) Frequency of spontaneous coils represented as the number of spontaneous coils per minute at 19 hpf. (H) Kinematic analysis of individual C-tail bends at 19 hpf. No differences were seen in the frequency or velocity of spontaneous coils. Error bars: s.e.m. Scale bar in A: 10 μm—CaPs; 50 μm—muscle and NMJ. (PDF) [file pone.0205255.s002.pdf]
