# Supplementary material for: 4.1Ba is necessary for glutamatergic synapse formation in the sensorimotor circuit of developing zebrafish
Source: PLoS One. 2018 Oct 4;13(10):e0205255. doi: 10.1371/journal.pone.0205255 (PMC6171929; doi:10.1371/journal.pone.0205255)
Supplement: S2 Table — (PDF) [file pone.0205255.s004.pdf]

S2 Table. Primers used for *in situ* hybridization

4.1Ba	Oligonucleotide Sequence (5' – 3')
Forward Primer	TAATACGACTCACTATAGGGCAGGAGGACGAGTCTGAAGC
Reverse Primer	AATTAACCCTCACTAAAGGGAGACGCAGATGCAGGAACAG

4.1Bb	Oligonucleotide Sequence (5' – 3')
Forward Primer	TAATACGACTCACTATAGGGCTGCTGCTGTCGGCGCGGAA
Reverse Primer	AATTAACCCTCACTAAAGGGCGATGTGTCGTCGTCATCACCGG
